# Supplementary material for: Rapid FFR: A rapid method for obtaining Frequency Following Responses
Source: bioRxiv. 2025 May 21:2025.05.20.655073. Preprint. [Version 1] doi: 10.1101/2025.05.20.655073 (PMC12139732; doi:10.1101/2025.05.20.655073)
Supplement: Supplement 1 [file media-1.pdf]

Appendices

A ABR measurements

Normal click-ABRs were obtained when wave V had latency values within 5.34 – 6.08 ms, which is ± 3 SDs around the mean for a 100 µs click at 70 dB nHL (=107.6 dB peSPL). The repetition time was kept at 11/s (Picton, Stapells, & Campbell, 1981).

B Additional Figures

For Experiment 1:

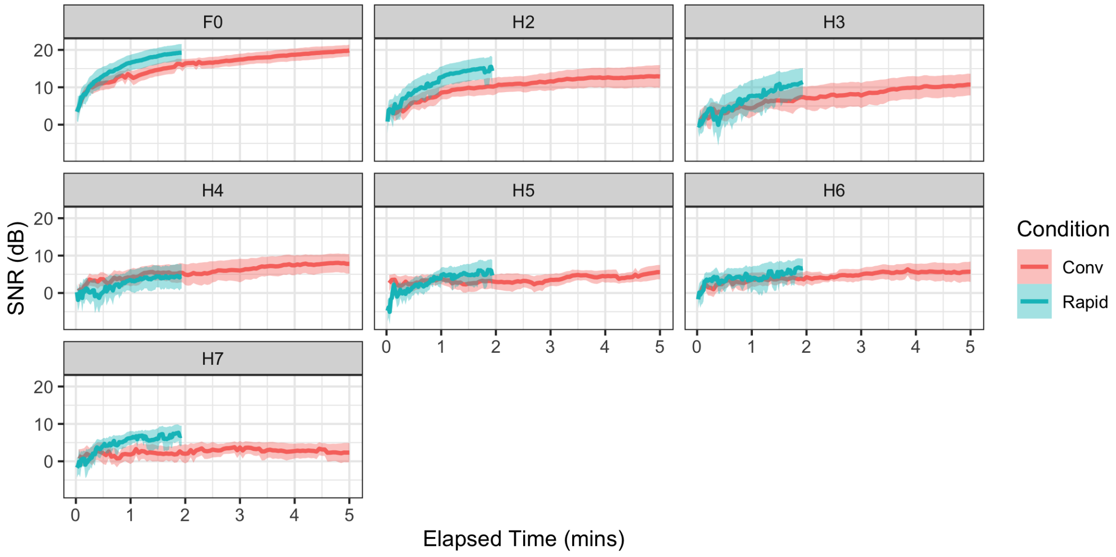

Figure 1: The growth in SNR for the first 7 harmonics of the recorded wave in the rapid and Conventional FFR as a function of elapsed time. Lines represent the mean across participants, and shaded areas show the 95 % Confidence intervals.

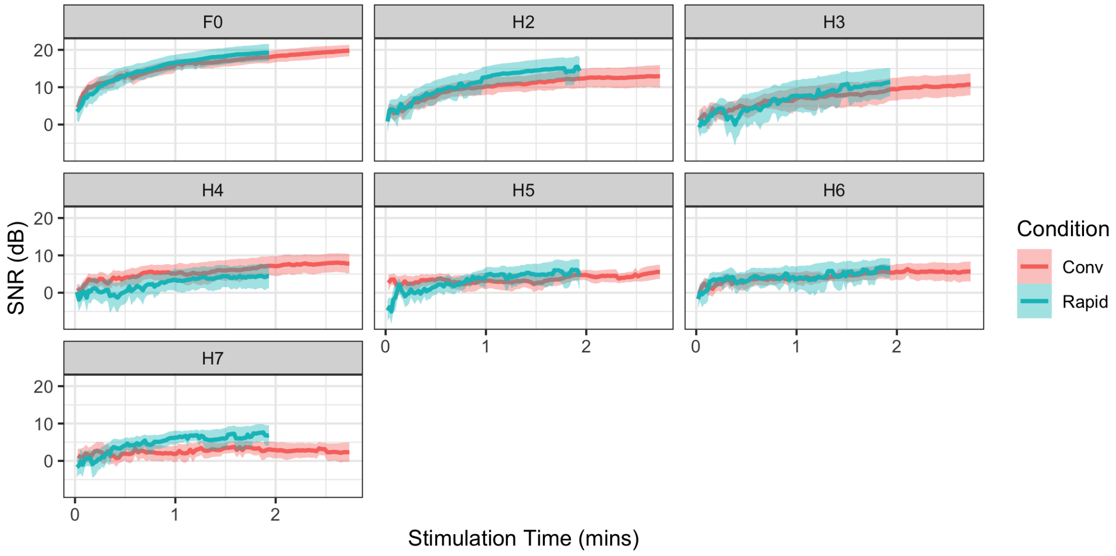

Figure 2: The growth in SNR for the first 7 harmonics of the recorded wave in the Rapid and Conventional FFR as a function of stimulation time. Lines represent the mean across participants, and shaded areas show the 95 % Confidence intervals.

For Experiment 2:

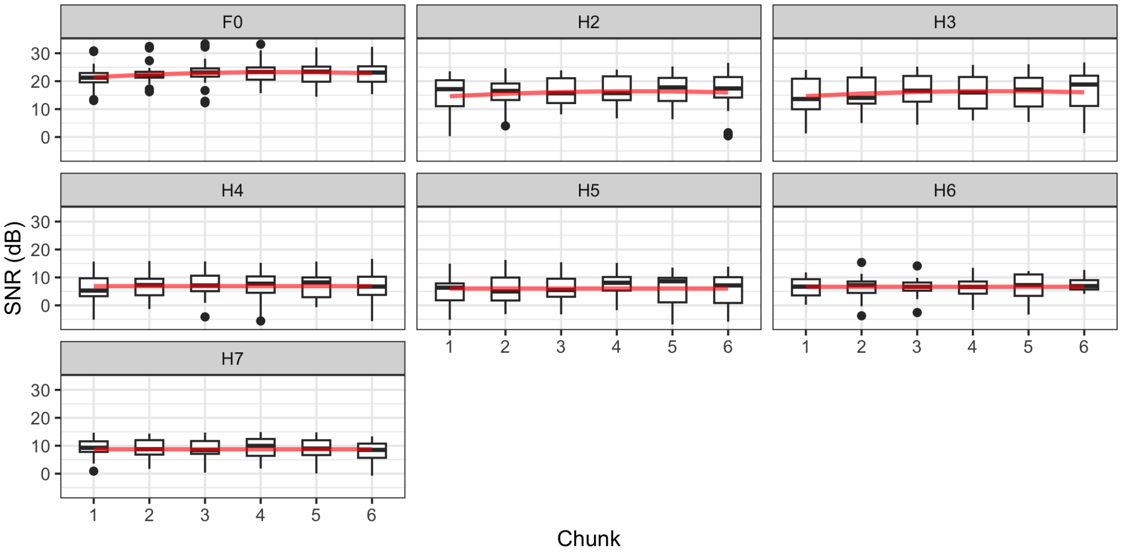

Figure 3: SNR levels of the Rapid FFR over an elapsed time of 9 minutes in each polarity. Here, we independently analyse 1/6th of the data in sequential chunks. Red lines show the values predicted by the linear mixed models.

C Additional Tables

factor	Estimate	Std. Error	dF	t-Value	p-Value
(Intercept)	20.545	1.947	222.667	10.551	<.001 *
Harmonic	-5.663	1.086	207.010	-5.217	<.001 *
I($Harmonic^2$)	.445	0.133	207.010	3.358	<.001 *
ConditionRapid	6.813	2.679	207.013	2.543	.001 *
Harmonic:ConditionRapid	-2.604	1.535	207.012	-1.696	.091
I($Harmonic^2$):ConditionRapid	.322	.187	207.010	1.715	.088

Table 1: Results for the linear mixed model which compares the SNR levels of the Rapid FFR with elapsed time-matched Conventional FFR. Significant p-values here (and in following tables) are marked by a '*'.

factor	Estimate	Std. Error	dF	t-Value	p-Value
(Intercept)	20.545	1.786	220.742	11.503	<.001 *
Harmonic	-5.663	.996	204.989	-5.686	<.001 *
I($Harmonic^2$)	.445	.122	204.988	3.660	.001 *
ConditionRapid	3.896	2.467	205.068	1.579	.116
Harmonic:ConditionRapid	-2.276	1.416	205.100	-1.608	.109
I($Harmonic^2$):ConditionRapid	.315	.173	205.137	1.821	.070

Table 2: Results for the linear mixed model comparing the SNR levels of the Rapid FFR with data volume-matched Conventional FFR.

Model	factor	Estimate	Std. Error	dF	t-Value	p-Value
F0-H3	(Intercept)	33.959	1.878	223.804	18.087	<.001 *
	Harmonic	-17.181	1.623	355.034	-10.589	<.001 *
	I($Harmonic^2$)	3.443	.402	355.035	8.576	<.001 *
	Chunk	1.389	.541	355.006	2.570	.011 *
	I($Chunk^2$)	-0.159	.076	355.002	-2.099	.037 *
H4-H7	(Intercept)	24.923	5.666	477.744	4.399	<.001 *
	Harmonic	-7.453	2.122	473.263	-3.513	<.001 *
	I($Harmonic^2$)	.734	.192	473.267	3.819	<.001 *

Table 3: Results for the linear mixed models which investigates the effect of *Chunk* on the SNR levels of the Rapid FFR.

Model	factor	Estimate	Std. Error	dF	t-Value	p-Value
F0-H3	(Intercept)	.287	.011	362.811	25.12	$<$.001 *
	Harmonic	-.184	.012	353.878	-14.76	$<$.001 *
	I($Harmonic^2$)	.034	.003	353.894	10.94	$<$.001 *
H4-H7	(Intercept)	.179	.019	482.2	9.278	$<$.001 *
	Harmonic	-.052	.007	477.4	-7.212	$<$.001 *
	I($Harmonic^2$)	.004	.001	477.4	6.791	$<$.001 *

Table 4: Results for the linear mixed models which investigates the effect of *Chunk* on the Signal levels of the Rapid FFR.

References

Picton, T. W., Stapells, D. R., & Campbell, K. B. (1981). Auditory evoked potentials from the human cochlea and brainstem. *The Journal of Otolaryngology.*, *9*, 1—41.
